# Effects of high-intensity gait training with and without soft robotic exosuits in people post-stroke: a *development-of-concept* pilot crossover trial

**DOI:** 10.1186/s12984-023-01267-9

**Published:** 2023-11-07

**Authors:** Franchino Porciuncula, Dheepak Arumukhom Revi, Teresa C. Baker, Regina Sloutsky, Conor J. Walsh, Terry D. Ellis, Louis N. Awad

**Affiliations:** 1 Department of Physical Therapy, Center for Neurorehabilitation, College of Health and Rehabilitation Sciences: Sargent College, Boston University, Boston, MA USA; 2 Department of Physical Therapy, Neuromotor Recovery Lab, College of Health and Rehabilitation Sciences: Sargent College, Boston University, Boston, MA USA; 3 Paulson School of Engineering and Applied Sciences, Harvard University, Cambridge, MA USA; 4 Department of Mechanical Engineering, Boston University, Boston, MA USA

**Keywords:** Exosuits, Robotics, Stroke, Gait

## Abstract

**Introduction:**

High-intensity gait training is widely recognized as an effective rehabilitation approach after stroke. Soft robotic exosuits that enhance post-stroke gait mechanics have the potential to improve the rehabilitative outcomes achieved by high-intensity gait training. The objective of this *development-of-concept* pilot crossover study was to evaluate the outcomes achieved by high-intensity gait training with versus without soft robotic exosuits.

**Methods:**

In this 2-arm pilot crossover study, four individuals post-stroke completed twelve visits of speed-based, high-intensity gait training: six consecutive visits of Robotic Exosuit Augmented Locomotion (REAL) gait training and six consecutive visits without the exosuit (CONTROL). The intervention arms were counterbalanced across study participants and separated by 6 + weeks of washout. Walking function was evaluated before and after each intervention using 6-minute walk test (6MWT) distance and 10-m walk test (10mWT) speed. Moreover, 10mWT speeds were evaluated before each training visit, with the time-course of change in walking speed computed for each intervention arm. For each participant, changes in each outcome were compared to minimal clinically-important difference (MCID) thresholds. Secondary analyses focused on changes in propulsion mechanics and associated biomechanical metrics.

**Results:**

Large between-group effects were observed for 6MWT distance (d = 1.41) and 10mWT speed (d = 1.14). REAL gait training resulted in an average pre-post change of 68 ± 27 m (p = 0.015) in 6MWT distance, compared to a pre-post change of 30 ± 16 m (p = 0.035) after CONTROL gait training. Similarly, REAL training resulted in a pre-post change of 0.08 ± 0.03 m/s (p = 0.012) in 10mWT speed, compared to a pre-post change of 0.01 ± 06 m/s (p = 0.76) after CONTROL. For both outcomes, 3 of 4 (75%) study participants surpassed MCIDs after REAL training, whereas 1 of 4 (25%) surpassed MCIDs after CONTROL training. Across the training visits, REAL training resulted in a 1.67 faster rate of improvement in walking speed. Similar patterns of improvement were observed for the secondary gait biomechanical outcomes, with REAL training resulting in significantly improved paretic propulsion for 3 of 4 study participants (p < 0.05) compared to 1 of 4 after CONTROL.

**Conclusion:**

Soft robotic exosuits have the potential to enhance the rehabilitative outcomes produced by high-intensity gait training after stroke. Findings of this *development-of-concept* pilot crossover trial motivate continued development and study of the REAL gait training program.

**Supplementary Information:**

The online version contains supplementary material available at 10.1186/s12984-023-01267-9.

## Background

Stroke is among the foremost causes of long-term disability in adults [1]. The persistence of slow walking speeds into the chronic phase of recovery impairs mobility across home and community environments [2]. Treatments that increase post-stroke walking speed are associated with improved long-distance walking [3], community mobility, and quality of life [4]. Thus, increasing walking speed is a major goal in stroke rehabilitation [5]. However, walking speed is often increased using compensatory strategies [6–8]. Indeed, substantial asymmetry in the propulsive forces generated by the paretic and nonparetic limbs is typical after stroke [9, 10], results in limited propulsion by the paretic limb when walking faster [11], and ultimately perpetuates patterns of intra- and inter-limb gait compensations [9, 10]. Targeted therapies that improve walking speed by restoring typical propulsive biomechanics [12–14] are needed to alter the common prognosis of persistent walking disability after stroke [6, 15].

Soft robotic exosuits are lightweight, textile-based robots that have emerged as viable devices for improving post-stroke walking speed by improving paretic limb function [16, 17]. We have shown that when powered on, soft robotic exosuits can immediately increase foot clearance during the paretic limb’s swing phase and forward propulsion during the paretic stance phase, resulting in improved propulsion symmetry, reduced gait compensations, and ultimately faster walking speeds and farther walking distances [16–19]. More recently, the rehabilitative potential of using soft robotic exosuits in the context of gait training has been examined. Specifically, our group has developed and studied the Robotic Exosuit-Augmented Locomotion (REAL) gait training program, which is a speed-based gait training program designed to exploit the exosuit’s ability to improve walking speed by improving paretic propulsion ability [20].

**Fig. 1:**
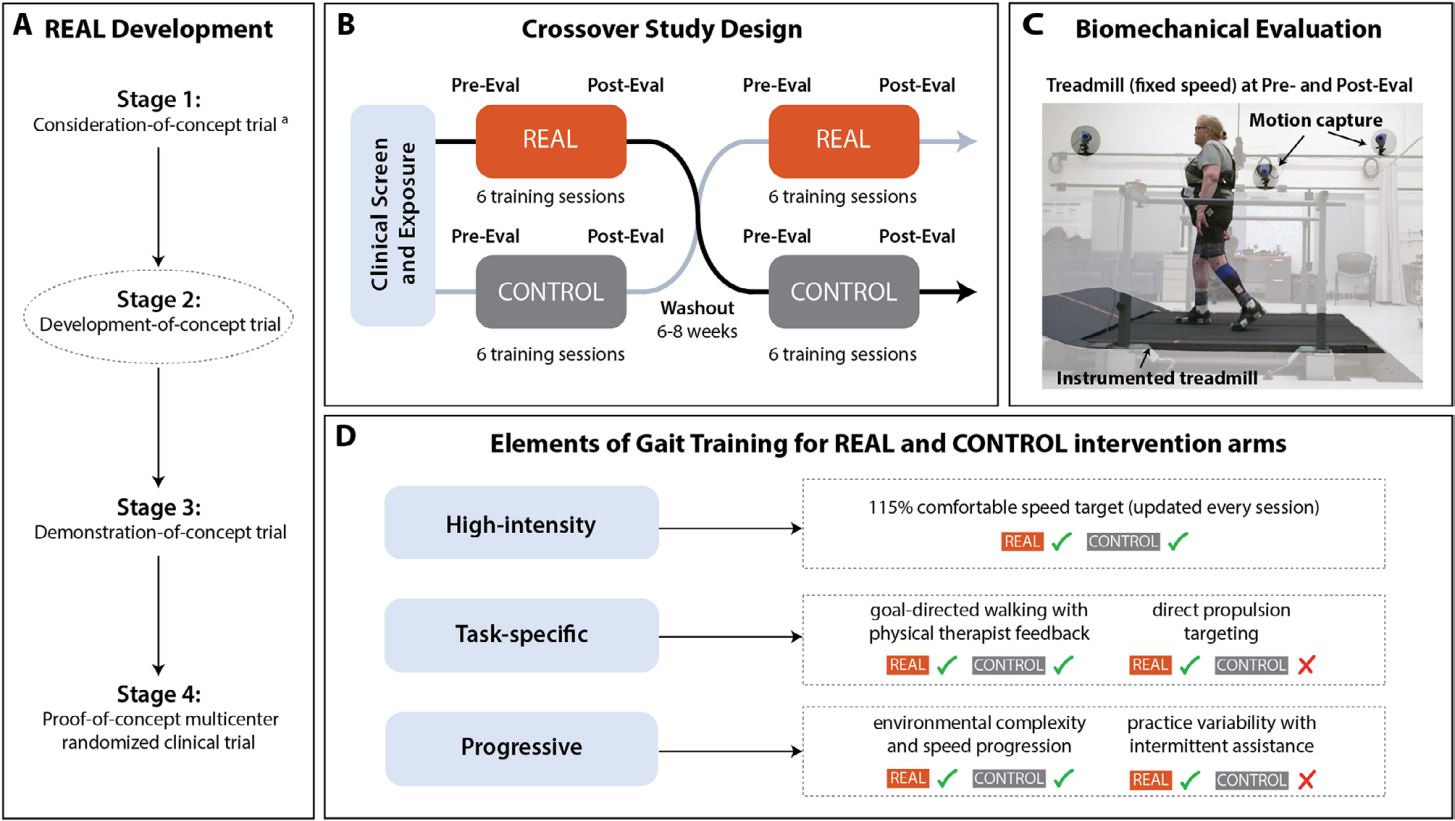
**Study design and key elements of REAL and CONTROL gait training programs**. **(A)** Staged clinical trial development approach used to develop and study the REAL gait training program. This trial is a stage 2, development-of-concept trial with **(B)** a crossover study design. **(C)** In addition to clinical outcomes of walking speed, as measured by the 10-meter walk test, and walking distance, as measured by the 6-minute walk test, biomechanical outcomes were evaluated at the pre- and post-training evaluation timepoints. **(D)** The key gait training elements for the REAL and CONTROL gait training programs. The programs were equivalent in terms of a progressive speed target provided within and across training visits, the provision of goal-directed feedback from a physical therapist, and training provided across settings. The programs differed in that the REAL gait program exploited the soft robotic exosuit to directly target propulsion impairment by way of intermittent (and continuous) gait assistance provided by the exosuit

The REAL gait training program aims to promote high-intensity, task-specific, and progressive rehabilitation with focus on improving both walking speed and gait quality (i.e., propulsion biomechanics). This dual focus contrasts with traditional gait training programs that may focus on *either* walking speed or gait quality. In examining novel robotic interventions such as REAL, it is critical to implement carefully planned successive stages of pilot studies to ensure sufficient optimization of the technology and intervention prior to conducting larger clinical trials [21]. This recommendation comes from synthesis of failed multi-center trials in the early decades of robotic assistive technologies, which suffered from suboptimal research pathways [21]. We thus utilize the strategic staging of pilot studies to examine and refine this new intervention (Fig. 1A). In a recently completed single-subject *consideration-of-concept* trial, we found that five sessions of REAL gait training elicited clinically meaningful improvements in walking function that were complemented by substantial improvements in propulsion biomechanics in the single subject that was the focus of the trial [20]. A follow-on pilot study with five individuals post-stroke similarly demonstrated that 18 sessions of REAL gait training produced clinically meaningful improvements in walking speed and distance, together with improved paretic limb kinematics [22]. These two preliminary studies of REAL gait training provide promising evidence for the rehabilitative value of soft robotic exosuits; however, without a control group, their results do not demonstrate the specific rehabilitative benefits of the exosuit technology. That is, speed-intensive gait training is known to facilitate high-intensity walking practice that is extremely effective in improving walking speed, distance, symmetry, and select kinematics on its own [23–25]. Therefore, the objective of this study was to evaluate, for the first time, the effects of REAL gait training in relation to the effects of dose-matched, high-intensity gait training without the exosuit technology. Comparing the rehabilitative effects of high-intensity gait training completed with versus without soft robotic exosuits is essential to inform development of subsequent clinical trials and, ultimately, to aid in the clinical translation of the soft robotic exosuit technology for post-stroke gait rehabilitation.

Another objective of this *development-of-concept* pilot crossover trial (see Fig. 1A and prior work [21] for pilot trial stages) was to examine three key implementation questions associated with the clinical deployment of the exosuit technology. First, we sought to understand the unique effects of REAL gait training on the rate of speed recovery. Though an evaluation of pre-to-post training outcomes is the standard for assessing treatment efficacy, such analyses may be affected by day-to-day variation in performance [26] and do not factor in the nonlinear processes involved in motor skill acquisition [27]. To lay early groundwork for understanding optimal dosing for future trials, we thus sought to evaluate the effect of soft robotic exosuits on the rate of speed recovery during high-intensity gait training. Second, to help establish proof-of-concept for the propulsion-targeted REAL gait training program, we sought to evaluate the effects of REAL training on propulsion biomechanics. Third, and finally, we sought to evaluate the importance of individualizing the exosuit assistive force profile over a multi-week training period. Prior work suggests that the individualization of robotic controller settings is necessary [28–30], and that the effects of robot-aided interventions are enhanced when controller settings match motor control objectives and individual user biomechanics [28, 31]. However, individualized tuning of exosuit force profiles has not yet been operationalized in the context of gait training with soft robotic exosuits, and it is not clear if an initial tuning session is sufficient, or if continued tuning over the course of the intervention period may be beneficial. The answers to these three implementation questions, taken together with the first controlled study of REAL gait training, will advance our understanding of the rehabilitative potential of soft robotic exosuits.

## Methods

### Participants

Individuals with stroke were enrolled into the study with the following inclusion criteria: stroke onset greater than 6 months (chronic phase), a score of 23 or greater in the Mini Mental State Exam, able to walk independently for 6 min, observable gait deficits, and neutral ankle dorsiflexion during standing. Individuals were excluded if they had physical, neurological, or medical co-morbidities that impair walking ability or if their comfortable walking speeds were faster than 1.0 m/s. Informed consent was obtained prior to study participation. All study procedures were approved by the Harvard University Institutional Review Board, which oversaw research activities conducted at both Harvard and Boston University.

### Exosuit

The soft robotic exosuit technology used in this study is described in detail in previous work [16, 18, 32]. In brief, the functional apparel worn on the paretic leg includes distal and proximal textile anchors used for cable-based force transmission. An actuator unit and battery pack are secured close to the body’s center-of-mass using a waist belt. Retraction of Bowden cables delivers assistive dorsiflexor or plantarflexor torques during specific targeted phases of the gait cycle. A position-based force controller [18] is used to provide plantarflexor assistance, where the maximum force and onset timing are selected based on the wearer’s response during a tuning procedure (see Sect. “Individualized tuning of exosuit force settings”). The dorsiflexor assistance settings are determined by a physical therapist through visual gait observation of foot clearance during swing [18]. Once tuned, these controller settings were fixed across all training days. Shoe-mounted inertial sensors enable gait detection and the delivery of the assistive forces in synchrony with the wearer’s gait. Moreover, these inertial sensors measured the number of steps taken during training visits [33], which was used as a measure of the volume of walking practice achieved during REAL gait training. The same set of inertial sensors were worn by study participants during the CONTROL gait training intervention period to enable comparison of the number of steps during the REAL versus CONTROL gait training periods.

### Study design

A crossover study design was implemented (Fig. 1B), where each study participant received both treatment interventions. The order of treatments was counterbalanced across study participants. Entry into the study began with an in-person clinical screen to determine eligibility and assess baseline clinical characteristics. A separate exposure session was conducted to acclimate and familiarize the study participant to the device. Next, a pre-training biomechanical evaluation was administered to measure baseline locomotor biomechanics (Fig. 1C) and to individualize exosuit settings to each user (Sect. “Individualized tuning of exosuit force settings”) prior to the onset of the first intervention period (Sect. “Intervention arms”). Each intervention period consisted of a total of 6 training sessions administered at a frequency of 3 sessions per week. A minimum frequency of 2 sessions per week was allowed to accommodate scheduling challenges. At the completion of the intervention period, a post-training biomechanical evaluation was performed, with procedures identical to the pre-training evaluation. A no-intervention washout period of 6 + weeks separated the intervention arms. All evaluation and training procedures were repeated for the second intervention arm.

### Intervention arms

#### Robotic exosuit augmented locomotion (REAL) training

A licensed physical therapist administered REAL gait training. Each training session consisted of 30 min of active walking, separated into five six-minute walking bouts of treadmill and overground walking. The maximum allowable heart rate for each training visit was set at 85% of the peak heart rate achieved during an electrocardiogram-guided graded exercise test that was completed prior to study enrollment.

**Fig. 2:**
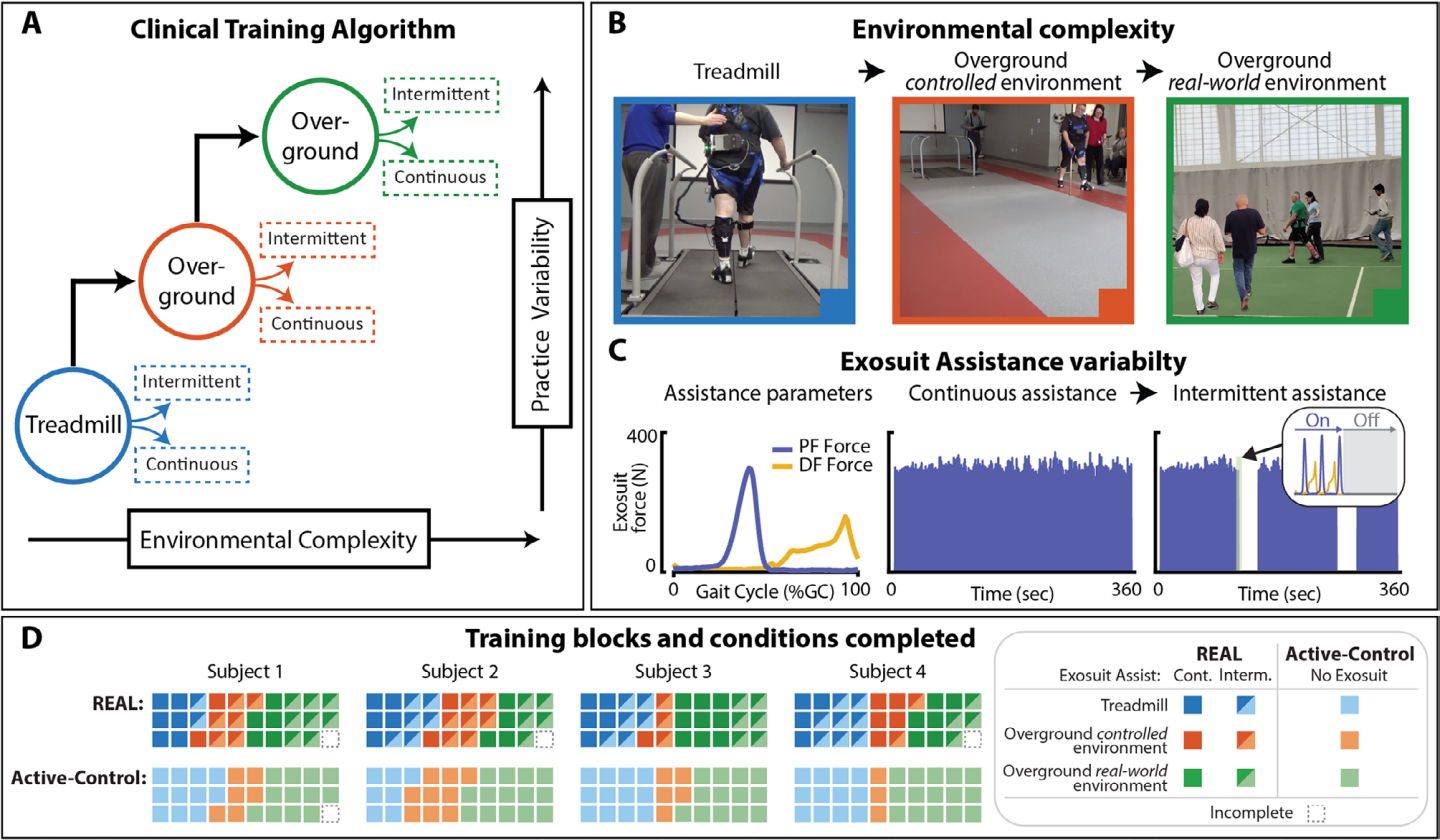
**Clinical Training Algorithm**. **(A)** Schematic of clinical training algorithm. **(B)** Training progression based on environmental complexity. **(C)** Training progression based on practice variability by manipulating scheduling of exosuit plantarflexion assistance from continuous to intermittent assistance. **(D)** Summary of completed walking trials by condition. *Abbreviations: Cont. – Continuous exosuit assistance (i.e., assistance provided every step). Interm. – Intermittent exosuit assistance (i.e., interleaving periods of walking with and without exosuit assistance)*

The REAL gait training program applies a clinical training algorithm that builds upon the methodology formulated and described in a precursor *consideration-of-concept* study [20], and draws on key principles of motor learning that are relevant in robot-assisted gait training: high-intensity, task-specificity, and individualization and progression (Fig. 2D).

1. High intensity training is implemented through a speed-based approach. Study participants are instructed to train at a walking speed set to 115% of their comfortable walking speed, as determined by a 10mWT performed at the start of every session.
2. Task-specific training is directed at addressing the problem of slow walking speeds at two levels. At the level of *Function* [34], 30 min of fast walking practice is provided during every treatment session. At the level of *Impairment* [34], the fast walking practice is structured to also promote enhanced push-off mechanics via targeted plantarflexor assistance from the soft robotic exosuit and verbal cues from the physical therapist, as needed. REAL gait training thus provides high-intensity, task-specific training centered on faster walking by way of plantarflexor-based propulsion.
3. Individualization and progression are implemented by systematically advancing environmental complexity and practice variability (Fig. 2A). Environmental complexity is progressed from simple and less complex environments (e.g., treadmill, overground walking inside the laboratory) to open and less predictable real-world environments (e.g., busy hallway, open space with busy pedestrian activity) (Fig. 2B). Practice variability is progressed by modulating the schedule of exosuit assistance, from uninterrupted continuous exosuit assistance to intermittent exosuit assistance. This was implemented through serial and alternating periods of power-on (120 s) and power-off (30 s) (Fig. 2C) during walking. During power-off, study participants were instructed to mimic their movements as when the device was powered on to encourage self-generated push-off mechanics during unassisted walking. Stepwise progression was determined by the study participant’s achievement of the set training speed of 115% of comfortable walking speed for each walking bout.

#### CONTROL: high-intensity gait training without exosuits

During the CONTROL intervention period, the same high-intensity gait training program as described for the REAL gait training program was implemented—with similar structure, dose, and progression algorithm—with the only exception being the soft robotic exosuit was not used during the treatment period.

### Individualized tuning of exosuit force settings

**Fig. 3:**
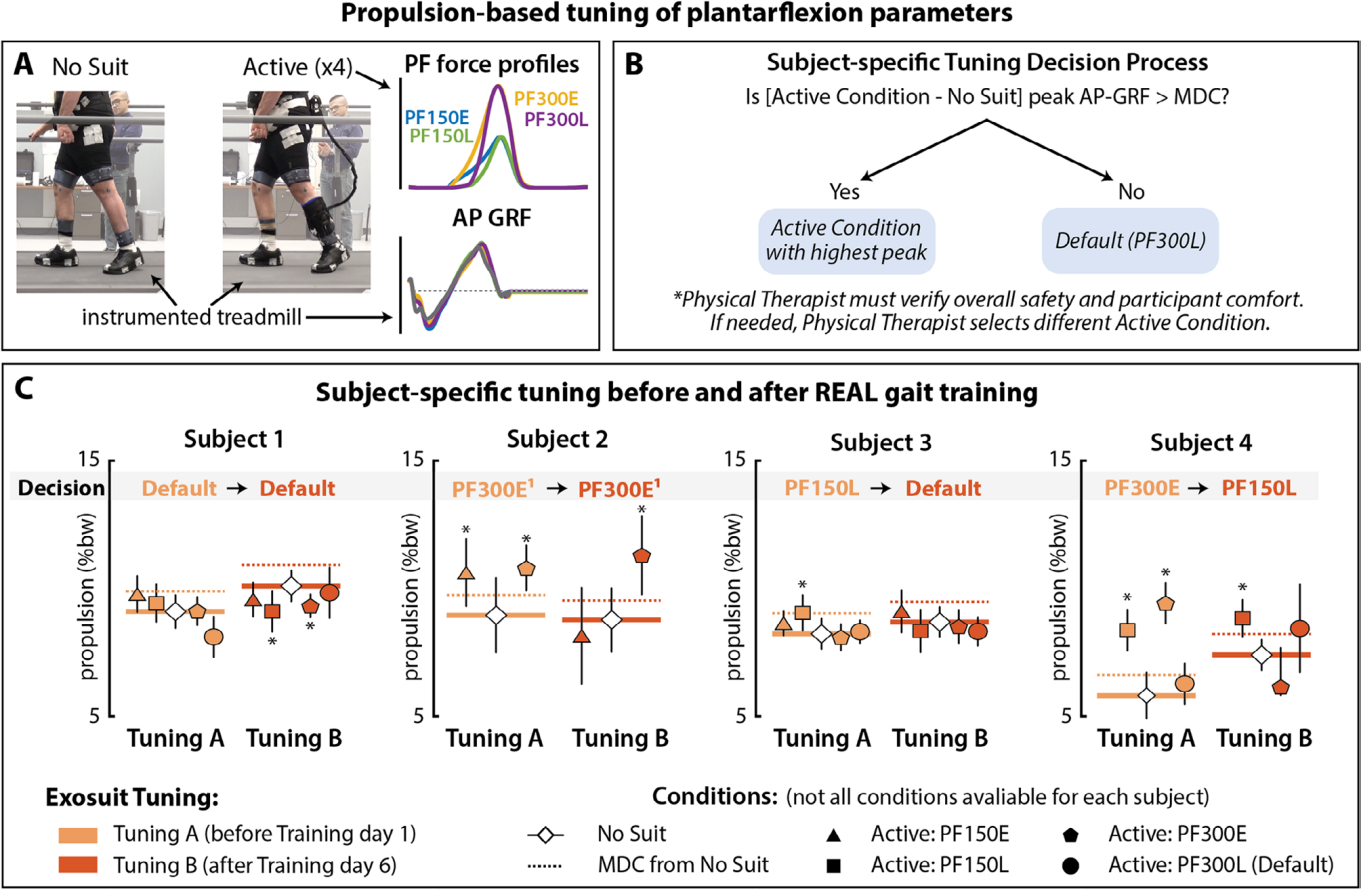
**Propulsion-based tuning of plantarflexion assistance parameters**. **(A)** Biomechanical examination of paretic propulsion across a range of plantarflexion (PF) force profiles. **(B)** Clinical decision process used to select the exosuit PF force setting to use during training. **(C)** Peak paretic propulsion for each tested exosuit force setting examined before and after REAL gait training. *Abbreviations: PF – Plantarflexor; AP GRF – Anterior-posterior ground reaction force; MDC – Minimal Detectable Change threshold*

The soft robotic exosuit’s plantarflexor force settings were tuned for each user prior to REAL gait training. The effects of four different plantarflexor force profiles were examined prior to the start of training (Fig. 3A): low (150 N) and high (300 N) exosuit force magnitudes were each implemented at early (10% of paretic single limb support) and late (90% of paretic single limb support) onset timings, abbreviated as PF150E, PF150L, PF300E, and PF300L, respectively. Due to technical issues, two participants (S2, S4) were not able to be administered all 4 profiles (See Fig. 3, C).

During the tuning procedure, study participants completed 2-minute trials on an instrumented treadmill (Bertec, Columbus, Ohio, USA) for each tested condition. The last 30 s of walking was used for data analysis. Testing was conducted at each study participant’s fast walking speed; walking speed was matched across all conditions. The user-individualized plantarflexor force profile selected for use during REAL training (see Fig. 3B) was the profile that generated the largest change in peak propulsion relative to baseline (i.e., no device) if the change was also greater than the minimal detectable change (MDC) of 0.80%BW [35]. If changes in propulsion were below the MDC, the PF300L profile was the default selection used during the gait training period. This default selection is based on the notion that higher force magnitudes may enable greater propulsion augmentation with continued exposure and practice, as well as prior evidence of better success with later plantarflexor assistance onset timings compared to earlier [36]. Finally, the physical therapist validated the setting per clinical observations of safety and user comfort, and recommended adjustments if needed. Once the exosuit settings were selected, these were fixed throughout the intervention period.

### Assessment and outcome measures

Before and after each treatment period, we evaluated study participants’ walking function based on the 10-Meter Walk Test (10mWT) and 6-Minute Walk Test (6MWT) (primary outcome measures) and propulsion biomechanics (secondary outcome measure), with all testing occurring without the soft robotic exosuit.

#### Clinical assessment: walking function outcomes

Short- and long-distance walking function were examined based on the 10mWT (maximum speed [m/s]) and the 6MWT (total distance [m]), respectively. To examine the rate of change in walking speed, 10mWT speed was also measured at the beginning of each training session.

#### Biomechanical assessment: propulsion outcomes

Locomotor biomechanics were examined during treadmill walking using an 18-camera motion capture system (Oqus, Qualysis, Gottenburg, Sweden; 200 Hz) and a force plate-embedded treadmill (Bertec, Columbus, Ohio, USA; 2000 Hz). Study participants walked on a treadmill set at their fast walking speed, defined as 115% of their comfortable walking speed on the treadmill. Walking assessments were speed-matched across the baseline and post-training timepoints. Out of 4 min of walking, the last 30 s of walking data were used for all analyses. From 3D motion analysis, we obtained peak paretic propulsion, defined as the peak of the anterior ground reaction force (percent body weight, [%bw]), and its associated metrics of ankle torque (Nm/kg), trailing limb angle (degrees) [37], and stride length (meter) [38]. Kinematic and kinetic data were filtered using a zero-lag low-pass 4th-order Butterworth filter with a 10 Hz-cutoff frequency. Biomechanical data were time-synchronized and normalized between sequential heel strikes measured using a 30 N vertical ground reaction force threshold.

### Statistical analyses

Baseline demographic and clinical information were summarized using descriptive statistics. Due to the potential for carryover effects arising from the crossover design of the study, we evaluated differences in baseline (pre-training) primary outcomes based on intervention arm using Wilcoxon Signed Rank test. Next, we examined for carryover effects based on order of interventions using Mann-Whitney U test [39]. For each study participant, changes in 10mWT speed and total distance walked during the 6MWT were first compared to published minimum clinically important difference (MCID) scores. For the 10mWT, published MCIDs range from a small change of 0.05 m/s to large substantial changes of 0.10 to 0.16 m/s [40–42]. For the 6MWT, published MCIDs range from a small change of 20 m to a substantial change of 50 m [42]. To help inform future clinical trial designs, the between-intervention effect on the 10mWT speed and total 6MWT distance was measured and reported using between-condition standardized effect sizes (Cohen’s *d*). Next, within-intervention effects were evaluated using pairwise comparisons of pre-to-post training data. The trajectory of speed recovery was then examined for each intervention using logarithmic linear regression. For the secondary propulsion biomechanics outcomes, analysis of pre-to-post training changes was done on an individual subject basis [35] using the Kruskall-Wallis test. For each analysis, alpha was set at 0.05, with Bonferroni adjustments for multiple comparisons.

**Table 1:** Baseline demographic and clinical information for study participants

Participant number	Paretic side	Stroke onset (y)	Sex	Age (y)	Height (cm)	Weight (kg)	CWS (m/s)	FMA-LE (of 34)	FGA (of 30)	ABC (of 100%)	SIS-p (of 40)
S1	Left	4.5	M	57	169	103	0.73	25	13	68%	22
S2	Right	3.8	M	60	174	94	0.74	26	12	49%	25
S3	Left	2.8	F	55	165	81	0.99	23	14	34%	21
S4	Right	4.0	M	62	174	82	0.74	20	12	90%	38
Average *± SD*:		3.8 *±* 0.7		59 *±* 3.1	171 *±* 4.4	90 *±* 10	0.80 *±* 0.13	24 *±* 2.6	12.7 *±* 1	60 *±* 24	26.5 *±* 8

**Note**: Baseline characteristics at study enrollment. *Abbreviations*: CWS: comfortable walking speed; FMA-LE: Fugl-Meyer Assessment-lower extremity; FGA: Functional Gait Assessment; ABC: Activities-specific Balance Confidence Scale; SIS-p: Stroke Impact Scale-Participation subsection

## Results

Four individuals with chronic stroke (59 ± 3 years, 171 ± 4 cm, 90 ± 10 kg, Table 1) participated in the study. As a group, study participants had moderate speed impairment; their median comfortable walking speed of 0.74 m/s corresponds to that of limited community ambulators [2].

### Safety and feasibility

Study participants completed the protocol without adverse events. On average, 98.35% and 99.18% of the 30 prescribed walking bouts for each intervention period were completed during the REAL and CONTROL intervention periods, respectively (Fig. 2,D). Each study participant progressed through all stages of the clinical training algorithm. The volume of steps measured during each intervention period were comparable. Marginal differences between the first and final training days were observed during each intervention period (see Supplementary Table B). By the final day of training, 2819 ± 585 steps were taken during REAL gait training and 2871 ± 584 steps were taken during CONTROL gait training.

### Data analysis of crossover design

Baseline walking function was comparable between groups, such that no baseline differences were noted for primary outcome measures of 6MWT (REAL: 326 ± 66 m, CONTORL: 345 ± 87, p = 0.47) and 10mWT (REAL: 1.08 ± 0.14 m, CONTORL: 1.15 ± 20, p = 0.27). Furthermore, there is no evidence of carryover effects based on primary outcome measures (6MWT, p = 0.67; 10mWT, p = 0.33).

**Table 2:** Changes in long- and short-distance walking by intervention arm

		CONTROL gait training				REAL gait training			
		Pre	Post	MCID	p-value	Pre	Post	MCID	p-value
10MWT	S1	1.08	1.10	**−**		0.92	1.02	↑	
	S2	1.08	1.05	**−**		1.07	1.14	↑	
	S3	1.44	1.40	**−**		1.27	1.37	↑	
	S4	0.99	1.08	↑		1.05	1.09	**−**	
	Group	1.15 (0.20)	1.16 (0.16)	**−**	0.759	1.08 (0.14)	1.16 (0.15)	↑	0.012
6MWT	S1	332	369	↑		268	355	↑↑	
	S2	292	338	↑		300	377	↑↑	
	S3	471	479	**−**		420	498	↑	
	S4	284	312	↑		314	342	↑↑	
	Group	344.8 (86.7)	374.5 (73.5)	↑	0.035	325.5 (65.9)	393 (71.5)	↑↑	0.015

Abbreviations: 10MWT = 10 m walk test; 6MWT = 6 min walk test; MCID: Minimal Clinical Important Difference;

Symbols: ↑: Small change ≥ MCID. ↑↑: Large change ≥ MCID. **−** : Change < MCID.

### Primary clinical outcomes

#### Effects on 10mWT speed and 6MWT distance

**Fig. 4:**
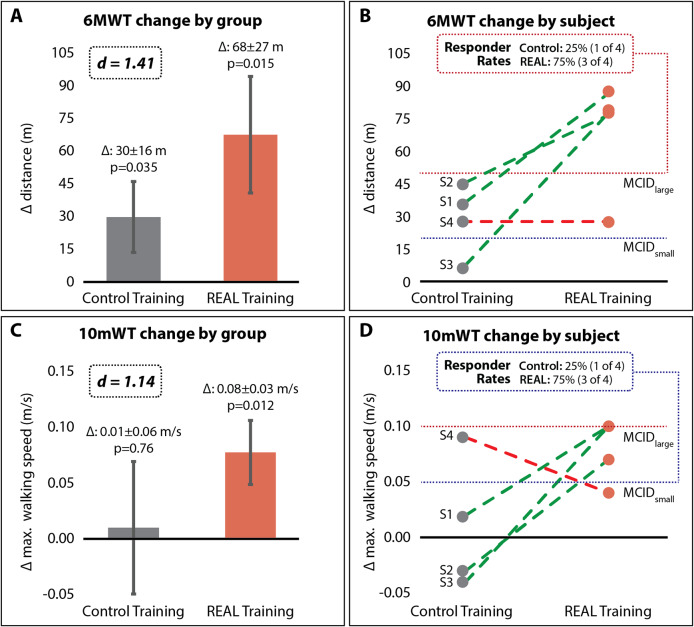
**Training-related effects on walking function outcomes**. **(A, B)** Group-level **(A)** and individual **(B)** changes based on 6-Minute Walk Test (6MWT) distance. **(C, D)** Group-level **(C)** and individual **(D)** changes based on 10-meter Walk Test (10mWT) maximum walking speed. Dashed red line = large MCID of 0.10 m/s [42]; Dashed blue line = small MCID of 0.05 m/s [42]

REAL gait training resulted in more 10mWT speed responders than CONTROL gait training (Fig. 4; Table 2). Specifically, after REAL gait training, three out of four study participants (S1, S2, S3) demonstrated small clinically meaningful changes (> MCID_small_ = 0.05 m/s) in 10mWT speed, compared to only one study participant following CONTROL gait training. Substantial changes (> MCID_large_ = 0.10) were not observed in either intervention period. A large between-intervention effect (Cohen’s *d* = 1.14) was observed between intervention periods, with REAL gait training resulting in an 8x larger gain in 10mWT speed compared to CONTROL gait training. More specifically, within-intervention analyses revealed a significant (p *<* 0.05) and clinically meaningful increase in maximum walking speed of 0.08 ± 0.03 m/s (i.e., + 7.2% average increase) after REAL gait training, whereas a nonsignificant (p *>* 0.05) and negligible change of 0.01 ± 0.06 m/s (i.e., + 0.9% average increase) was observed after CONTROL gait training.

Similarly, REAL gait training resulted in more 6MWT distance responders than CONTROL gait training (Fig. 4; Table 2). Specifically, after REAL gait training, three out of four study participants (S1, S2, S3) demonstrated large clinically meaningful changes (> MCID_large_ = 50 m) and one study participant (S4) demonstrated a small clinically meaningful change (> MCID_small_ = 20 m) in 6MWT distance. In contrast, after CONTROL gait training, no study participants surpassed the MCID_large_ threshold and three of four study participants surpassed the MCID_small_ threshold. A large between-intervention effect (Cohen’s *d* = 1.41) was observed between intervention periods, with REAL gait training resulting in a > 2.25x gain in 6MWT distance compared to CONTROL gait training. More specifically, within-intervention analyses revealed significant improvements in 6MWT distance during both intervention periods (p’s *<* 0.05); however, a large clinically-meaningful average change of 68 ± 27 m (i.e., + 20.7% average increase) was achieved following REAL gait training, whereas only a small clinically-meaningful average change of 30 ± 16 m (i.e., + 8.6% average increase) was observed following CONTROL gait training.

**Fig. 5:**
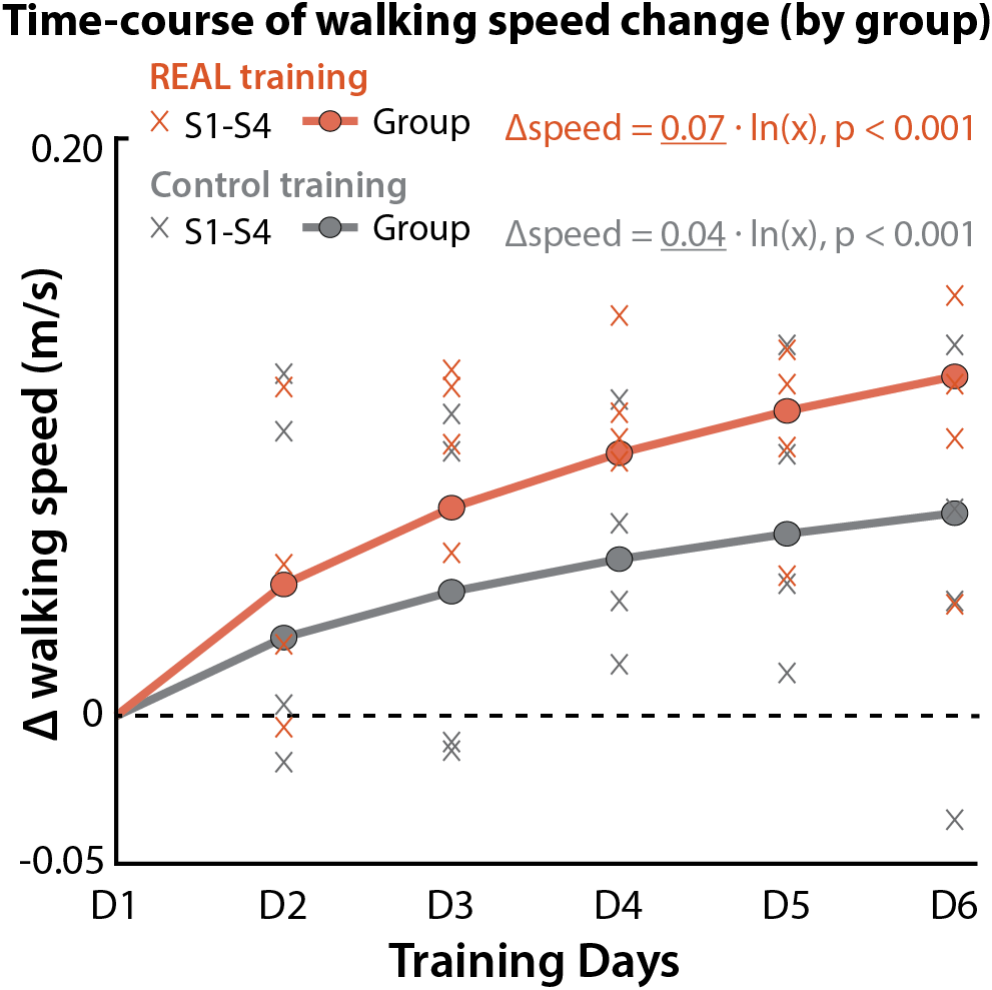
**Trajectory of speed change by intervention arm**. Change in walking speed across training sessions (Day 1 (D1) to Day 6 (D6) for each intervention arm

#### Effects on rate of change in walking speed

REAL gait training resulted in a larger rate of change in walking speed over the training period compared to CONTROL gait training (Fig. 5). Curve fitting of session-by-session walking speeds using the natural logarithmic function for the REAL gait training and CONTROL gait training intervention periods revealed a 1.75 higher exponential change in walking speed during the REAL gait training period (*y* = 0.07*ln*(*x*), *R*^2^ = 0.54, *RMSE* = 0.03 *m/s, p <* 0.001, 95%CI [0.05, 0.08]) compared to the CONTROL gait training period (*y* = 0.04*ln*(*x*); *R*^2^ = 0.08, *RMSE* = 0.05 *m/s, p <* 0.001, 95%CI [0.02, 0.06]).

### Secondary biomechanical outcomes

#### Effects on peak paretic propulsion

REAL gait training resulted in more propulsion responders than CONTROL gait training (Table 3). Specifically, after REAL gait training, three out of four study participants (S1, S2, S3) demonstrated significantly improved peak paretic propulsion (p < 0.05). In contrast, CONTROL gait training resulted in highly mixed effects: whereas one study participant (S3) significantly improved paretic propulsion (p < 0.05), another study participant (S1) significantly worsened paretic propulsion (p *<* 0.05), and two study participants (S2, S4) had negligible and non-significant changes (p’s *>* 0.05) in paretic propulsion.

#### Effects on propulsion-related biomechanics

REAL gait training resulted in more study participants improving key propulsion-driving biomechanical variables compared to CONTROL gait training. Specifically, after REAL gait training, three study participants (S1, S3, S4) significantly increased their paretic trailing limb angle and stride length, with two of these study participants (S1 and S3) concurrently increasing their plantarflexion torque (p’s < 0.05). Conversely, after CONTROL gait training, two study participants significantly increased their paretic trailing limb angle and stride length (S2 and S3), with one of these study participants (S2) concurrently increasing their plantarflexor torque (p’s *<* 0.05). A third study participant increased just their plantarflexor torque (S4) (p *<* 0.05). An exploratory examination of changes in overground propulsion biomechanics was conducted for a subset of 2 study participants (see, Supplementary Table B).

### Propulsion-based tuning of exosuit force settings

The propulsion-based exosuit tuning procedure conducted prior to engaging in the REAL gait training program (Fig. 3C) resulted in three study participants demonstrating meaningful improvements in peak propulsion with user-individualized exosuit force profiles: S2 - PF300E, S3 - PF150L, and S4 - PF300E. These exosuit assistance profiles were used for REAL gait training. One study participant (S1) failed to match to any specific exosuit force profile; the default profile of PF300L was thus used for REAL gait training. The supervising physical therapist confirmed that these user-individualized profiles were safe and comfortable, requiring no further adjustment. At the completion of REAL gait training, the same tuning procedures were repeated and revealed an alteration in individualized exosuit profiles for two participants: S3 - PF300L (from PF300E) and S4 - PF150L (from PF300E). The two other study participants, S1 and S2, demonstrated no needed changes in their exosuit profiles.

**Table 3:** Comparison of training-related effects on propulsion biomechanics and related metrics

		CONTROL			REAL		
		Pre	Post	p-value	Pre	Post	p-value
**Peak Propulsion**	S1	9.77 (0.70)	8.81 (1.21)	**0.001**	9.10 (1.30)	10.10 (1.22)	**0.003**
	S2	6.63 (2.68)	7.36 (3.01)	> 0.05	8.95 (2.93)	8.77 (2.49)	> 0.05
	S3	9.84 (1.65)	11.08 (1.97)	**0.030**	8.22 (1.19)	8.69 (1.18)	**0.025**
	S4	7.09 (2.37)	7.00 (2.79)	> 0.05	5.81 (1.80)	7.40 (1.19)	**0.003**
**Plantarflexor Torque**	S1	1.00 (0.07)	1.04 (0.07)	> 0.05	0.85 (0.08)	0.92 (0.08)	**0.001**
	S2	1.40 (0.19)	1.46 (0.19)	**0.036**	1.44 (0.28)	1.36 (0.10)	> 0.05
	S3	1.44 (0.15)	1.44 (0.08)	> 0.05	1.39 (0.10)	1.43 (0.13)	**0.030**
	S4	0.93 (0.09)	1.04 (0.19)	**0.033**	0.88 (0.16)	0.96 (0.22)	> 0.05
**Trailing limb angle**	S1	23.93 (0.66)	23.86 (0.69)	> 0.05	21.39 (1.16)	23.10 (0.56)	**< 0.001**
	S2	16.62 (1.91)	18.55 (2.57)	**0.011**	17.59 (1.84)	17.76 (1.05)	> 0.05
	S3	22.77 (2.32)	26.51 (1.60)	**< 0.001**	21.00 (2.18)	22.42 (2.17)	**0.005**
	S4	14.48 (2.07)	14.10 (2.96)	> 0.05	12.88 (1.88)	14.08 (1.87)	**0.010**
**Stride length**	S1	0.99 (0.04)	0.99 (0.03)	> 0.05	0.86 (0.06)	0.93 (0.03)	**< 0.001**
	S2	1.03 (0.05)	1.16 (0.05)	**< 0.001**	1.15 (0.07)	1.17 (0.04)	> 0.05
	S3	0.90 (0.11)	0.99 (0.07)	**< 0.001**	0.82 (0.06)	0.92 (0.06)	**< 0.001**
	S4	0.97 (0.04)	0.96 (0.05)	> 0.05	0.93 (0.04)	0.97 (0.04)	**0.010**

Measurements of paretic propulsion function during speed-matched treadmill walking at Pre- and Post-Training evaluation timepoints for each intervention arm. Significant within-group differences in **boldface** (p < 0.05)

## Discussion

In this *development-of-concept*, pilot crossover trial, we evaluated the rehabilitative value of soft robotic exosuits by comparing high-intensity gait training with versus without soft robotic exosuits. Consistent with the literature on high-intensity gait training [43–45], though both interventions improved post-stroke walking function, particularly 6-minute walk test distance, we found that Robotic Exosuit Augmented Locomotion (REAL) gait training resulted in substantially greater improvements in 6-minute walk test distance, larger and more clinically-meaningful gains in walking speed, a faster rate of speed increase across training sessions, and improvements in gait propulsion that were not seen with high-intensity gait training alone.

### Soft robotic exosuits enhance the outcomes observed with high-intensity gait training

Soft robotic exosuits, when used in the context of the REAL gait training program, can amplify the effects of high-intensity gait training on post-stroke walking speed and distance. To enhance the rigor of this initial controlled evaluation of the REAL intervention, we studied the effects of dose-matched and comparable interventions to allow direct comparison between REAL and CONTROL gait training. Both the REAL and CONTROL interventions provided gait training at high-intensity and with a high-volume of steps. More specifically, study participants received 30 min cumulative of fast walking practice every session, resulting in between 2600 and 2900 steps per session during both interventions. This volume of walking practice heavily contrasts with usual care where the typical number of steps completed per session are extremely low (i.e., an average of 370 steps per therapy session [46]). The high intensity and volume of walking practice delivered during this pilot crossover trial helps explain why both the REAL and CONTROL interventions achieved meaningful gains in walking function with only six training sessions, though the CONTROL intervention produced markedly smaller improvements in 6-minute walk test distance and 10mWT speed when compared to the REAL intervention.

In contrast to the current study’s training dose of 6 training sessions per intervention arm, a recently completed single-arm pilot study of REAL gait training conducted by Shin et al. demonstrated that 18 sessions of REAL gat training resulted in even larger improvements in 10mWT walking speed than observed in this study, with an improvement of 0.21 m/s [22]. This suggests that larger speed benefits can be appreciated with a greater dose of REAL gait training. We posit that the distinguishing element in REAL is the emphasis on both walking speed and gait propulsion; the soft robotic exosuit’s enhancement of paretic propulsion appears to enrich the speed-based, high-intensity gait training program at the core of REAL gait training. That is, by exploiting the soft robotic exosuit’s instantaneous benefits on propulsion [18, 36] and speed [16], the quality of walking practice provided during REAL gait training may enable greater therapeutic effects. These promising findings arising from optimization of the device and the deliberate integration of motor learning principles in the rehabilitation program offer a working gestalt for other propulsion-targeting wearable systems to leverage, both during device development and clinical deployment. Further development and study of the REAL gait training program, and similar rehabilitation programs developed for other next-generation wearable technologies, is warranted.

### Learning rates are faster with the targeted gait training provided with soft exosuits

Most clinical trials in stroke rehabilitation examine training effects, learning, and change in motor behavior based on pre-post assessments [26, 27]. However, before and after measurements alone do not elucidate the processes and mechanisms of learning [27, 47]. In this study, we examined the trajectory of speed changes across sessions to understand whether time scales of speed recovery differed by intervention. Our findings revealed that the acquisition of walking speed significantly increased over the course of gait training for both interventions. The buildup in speed over time suggests that the speed-based intervention has an additive effect on speed recovery. However, when comparing interventions, there is a notable steeper rise in speed acquisition in favor of using soft robotic exosuits compared to the CONTROL intervention. The faster rate of improvement during REAL concurs with the larger and more substantial pre-post changes in short- and long-distance walking function improvements. The performance equation curves generated from the trial data may assist with the design of future dose-response studies. Indeed, the REAL performance equation curve concurs with the training-related speed changes observed in the aforementioned pilot study by Shin et al. [22].

### Proof-of-concept based on biomechanical changes on propulsion function

Soft robotic exosuits are propulsion-targeting devices [18, 36]. In this pilot crossover trial, we examined participant-specific changes in paretic propulsion and its determinants of paretic plantarflexor torque and trailing limb angle [37]. The participant-specific responses observed are consistent with the premise of this study. Specifically, 3 participants (S1, S3, S4) demonstrated significant increases in propulsion following REAL gait training. These same study participants demonstrated biomechanically-aligned patterns of improvement in trailing limb angle (S1, S3, S4) and plantarflexor torque (S1, S3) as would be expected with their propulsion improvements. This contrasts with the variable responses following CONTROL gait training, where we observed mixed responses of improvement (S3), reduction (S1), and absent change (S2, S4) in paretic propulsion. Moreover, following CONTROL intervention, variable biomechanical changes were observed with improvements in plantarflexor torque for one participant (S2) and improvements in trailing limb angle for two (S2, S3). The seemingly varied pairing of propulsion, trailing limb angle, and plantarflexor torque changes in the CONTROL intervention period contrasts heavily with the biomechanically-consistent changes observed following the REAL intervention.

It is noteworthy that the propulsion changes observed in this study were markedly smaller compared to the substantial changes observed in the clinical outcomes of walking speed and walking distance. It is possible that our method of examining locomotor biomechanics (i.e., speed-matching of treadmill conditions) may have dampened the measurable changes in propulsion produced by the intervention period. That is, because propulsion and speed are co-dependent, by fixing the post-training treadmill evaluation speed to the (slower) treadmill evaluation speed used at baseline, the forced slower walking speeds at post-training may have masked a greater ability to produce propulsive forces by our study participants. Indeed, in a subset of 2 study participants (S3, S4) who we were able to add overground walking biomechanics evaluations (see Supplementary Table 2), we measured substantially larger increases in paretic propulsion following REAL gait training during the overground evaluations (∆s: 2.95%bw and 6.5%bw for S3 and S4, respectively) than what was observed during their treadmill evaluations (∆s: 0.47%bw and 1.59%bw, respectively). Moreover, these marked changes in overground propulsion function following REAL gait training contrast heavily with the much smaller changes in overground propulsion function observed following CONTROL gait training (∆s: 2.02%bw and 0.81%bw, respectively). Future trials designed to evaluate the training-induced effects on propulsion biomechanics should consider testing with updated speeds on the treadmill (in addition to or instead of baseline-matched speeds), or evaluating propulsion biomechanics during unconstrained overground walking.

### Exosuit force settings matters

The severity of walking impairment after stroke is variable and dependent on multiple factors ranging from cardiovascular fitness, neuromotor impairments, and limb mechanics [10, 48, 49]. The success of exosuit devices thus depends on how well the robotic controller settings match user-specific needs [28–30, 50]. Optimizing the device settings to the user is an important consideration of wearable systems to maximize the benefit to the user [51], however tuning procedures are often time-intensive, making them less suitable for those who have neuromotor impairments and are susceptible to fatigue. In the current study, we individualized the exosuit force profiles used during exosuit-augmented gait training based on direct measurement of the effect of different exosuit force profiles on the paretic limb’s propulsion output (i.e., the biomechanical target of the exosuit’s plantarflexor force assistance). To simplify the tuning procedure for future clinical use, we evaluated up to 4 pre-fixed settings (instead of a more incremental scale). We found differential responses to exosuit force profiles in 3 study participants (S1, S3, S4), which suggests that these force settings have distinct effects on the user. Further, there were instantaneous gains in propulsion that exceeded MDC thresholds in 3 study participants (S2, S3, S4) at baseline, 2 of which also demonstrated therapeutic improvements in propulsion after training. Altogether, these results suggest that immediate and clinically meaningful enhancements in paretic propulsion can be achieved with a properly tuned device, and that these immediate effects may have implications on the therapeutic outcomes resulting from training. Finally, the changes in optimal exosuit profiles at the end of training as seen in 2 out of 4 participants raise considerations for re-tuning at different timepoints in a multi-week training program.

## Limitations

Our study has several limitations. First, this preliminary pilot study included a small number of study participants. While limited in sample size, the intent of the crossover pilot study was to evaluate the unique effects of the exosuit-augmented intervention on a small number of study participants to understand individual responses and inform future, larger clinical trials. Carefully planned small pilot studies such as the current study serve as foundation for larger studies in clinical and translational sciences [52]. Second, while within-group pre-to-post training analyses were conducted to examine within-group differences for the primary clinical outcome measures, the comparisons between the REAL and CONTROL interventions were performed in a descriptive manner (in addition to reporting between-intervention effect sizes). Further, within-subject statistical analyses were performed at an individual-level for secondary outcomes. We opted to not perform between-group statistical analyses for the primary and secondary outcomes due to the limited sample size of this development-of-concept trial. Finally, the crossover nature of the study does not eliminate completely the possibility for carryover effects onto the following intervention arm. We minimized this potential confound by providing a washout period that was at least 3 times the duration of the first intervention period, and we administered the intervention arms in alternating sequence. Further, our analysis revealed no statistical differences in baseline long- and short-distance walking function between interventions. Baseline values of walking function for each intervention were at levels that were well-below the age normative range [51, 52], which is indicative of substantial potential for improvement for each intervention arm. That is, neither intervention period was constrained by ceiling effects. Moreover, there was no statistical evidence of carryover effects based on sequence of intervention.

## Conclusion

In this 2-arm, pilot crossover study including four individuals with chronic post-stroke hemiparesis, soft robotic exosuits were safe to use during high-intensity gait training and were found to enable substantial and expedient recovery of short- and long-distance walking function compared to dose-matched high-intensity gait training without soft robotic exosuits. This *development-of-concept* trial supports further development and study of Robotic Exosuit Augmented Locomotion (REAL) gait training.

### Electronic supplementary material

Supplementary table for Tables (PDF 38 kb)

## Data Availability

Data will be made available upon request.

## Abbreviations

6MWT: 6-minute walk test
10mWT: 10-m walk test
PF150E: Plantarflexion force of 150 N with early timing onset
PF150L: Plantarflexion force of 150 N with late timing onset
PF300E: Plantarflexion force of 300 N with early timing onset
PF300E: Plantarflexion force of 300 N with late timing onset
MCID: Minimal clinically important difference
MDC: Minimal detectable change
REAL: Robotic Exosuit Augmented Locomotion
TLA: Trailing limb angle
