# Supplementary material for: Effects of high-intensity gait training with and without soft robotic exosuits in people post-stroke: a development-of-concept pilot crossover trial
Source: J Neuroeng Rehabil. 2023 Nov 7;20:148. doi: 10.1186/s12984-023-01267-9 (PMC10629136; doi:10.1186/s12984-023-01267-9)
Supplement: Supplementary file 1 — Supplementary table for Tables (PDF 38 kb) [file 12984_2023_1267_MOESM1_ESM.docx]

**Supplementary**

**Supp.Table A** Total step counts achieved at the start and end of training intervention

|  |  | Step Counts D1 | Step Counts  D6 |  |
| --- | --- | --- | --- | --- |
| REAL | S1 | 2214 | 2312 |  |
|  | S2 | 2262 | 2752 |  |
|  | S3 | 3468 | 3654 |  |
|  | S4 | 2452 | 2560 |  |
|  | Ave (SD) | 2599 (588) | 2819 (585) |  |
|  |  |  |  |  |
| Control | S1 | 2380 | 2432 |  |
|  | S2 | 2352 | 2774 |  |
|  | S3 | 3646 | 3722 |  |
|  | S4 | 2492 | 2558 |  |
|  | Ave (SD) | 2718 (621) | 2872 (584) |  |

D1 = training day 1, D6 = training day 6

**Supp.Table B** Supplementary Table: Changes in propulsion function during overground walking

|  |  | **Peak propulsion** | | | **Ankle torque** | | | **Trailing limb angle** | | |
| --- | --- | --- | --- | --- | --- | --- | --- | --- | --- | --- |
|  |  | Pre | Post | p=value | Pre | Post | p-value | Pre | Post | p-value |
| REAL | S3 | 13.20 (2.03) | 16.16 (1.64) | **<0.001** | 1.41 (0.11) | 1.40 (0.06) | >0.05 | 26.74 (1.09) | 28.34 (0.65) | **<0.001** |
|  | S4 | 9.16 (2.38) | 15.66 (3.95) | **0.049** | 1.04 (0.03) | 1.37 (0.10) | **0.049** | 32.61 (11.49) | 31.94 1.33) | >0.05 |
|  |  |  |  |  |  |  |  |  |  |  |
| Control | S3 | 14.28 (2.17) | 16.30(1.41)^∗^ | **0.008** | 1.68 (0.63) | 1.48 (0.26) | >0.05 | 28.81 (1.72) | 29.58 (1.68) | >0.05 |
|  | S4 | 13.01 (2.39) | 13.82 (3.91) | >0.005 | 1.22 (0.07) | 1.29 (0.21) | >0.05 | 30.74 (8.37) | 31.03 (5.99) | >0.05 |
|  |  |  |  |  |  |  |  |  |  |  |

Tx = intervention arm; Time = assessment time point;

* = significant between Pre and post-eval within subject (K-W test, p*<*0.05)
